# Performance Analysis of Multihop Full-Duplex NOMA Systems with Imperfect Interference Cancellation and Near-Field Path-Loss

**DOI:** 10.3390/s23010524

**Published:** 2023-01-03

**Authors:** Lam-Thanh Tu, Van-Duc Phan, Tan N. Nguyen, Phuong T. Tran, Tran Trung Duy, Quang-Sang Nguyen, Nhat-Tien Nguyen, Miroslav Voznak

**Affiliations:** 1 Communication and Signal Processing Research Group, Faculty of Electrical and Electronics Engineering, Ton Duc Thang University, Ho Chi Minh City 756000, Vietnam; 2 Faculty of Automotive Engineering, School of Engineering and Technology, Van Lang University, Ho Chi Minh City 710000, Vietnam; 3 Faculty of Electrical Engineering and Computer Science, VSB—Technical University of Ostrava, 70800 Ostrava, Czech Republic; 4 Wireless Communications Research Group, Faculty of Electrical and Electronics Engineering, Ton Duc Thang University, Ho Chi Minh City 756000, Vietnam; 5 Posts and Telecommunications Institute of Technology, Ho Chi Minh City 710000, Vietnam; 6 Science and Technology Application for Sustainable Development Research Group, Ho Chi Minh University of Transport, Ho Chi Minh City 717000, Vietnam

**Keywords:** full-duplex communications, multihop communications, NOMA, outage probability, potential throughput

## Abstract

Outage probability (OP) and potential throughput (PT) of multihop full-duplex (FD) nonorthogonal multiple access (NOMA) systems are addressed in the present paper. More precisely, two metrics are derived in the closed-form expressions under the impact of both imperfect successive interference cancellation (SIC) and imperfect self-interference cancellation. Moreover, to model short transmission distance from the transmit and receive antennae at relays, the near-field path-loss is taken into consideration. Additionally, the impact of the total transmit power on the performance of these metrics is rigorously derived. Furthermore, the mathematical framework of the baseline systems is provided too. Computer-based simulations via the Monte Carlo method are given to verify the accuracy of the proposed framework, confirm our findings, and highlight the benefits of the proposed systems compared with the baseline one.

## 1. Introduction

Non-orthogonal multiple access (NOMA) along with other advanced technologies such as deep learning [1,2,3], reconfigurable intelligent surfaces (RISs) [4,5,6,7], and tools from stochastic geometry (SG) [8,9,10,11] have been considered key-driven technologies for cellular networks beyond 5G, i.e., 5G-Advanced. More precisely, by allowing simultaneous transmit multiple signals within the same resource blocks (frequency and time) via different power levels and/or codes, NOMA can significantly enhance the spectral efficiency (SE) of the networks [12,13]. Additionally, NOMA can effortlessly combine with other techniques to further enhance system performance. One of the favorite combinations is to deploy full-duplex (FD) communications [14,15]. In fact, by concurrently transmitting and receiving signals, FD communications, theoretically, doubly improve spectral efficiency [16]. Although both FD and NOMA greatly facilitate the SE of the wireless networks, they suffer from the low signal-to-interference-plus-noise ratio (SINR) owing to strong self-interference [17,18]. Hence, to truly attain benefits from both NOMA and FD, ameliorating the SINR is mandatory, one of the promising solutions is to utilize multihop communications that improve the SINR by shortening the transmission distance [19,20]. Particularly, if the transmission distance is compressed, the intended signals are improved while the self-interference does not remarkably change thus scaling up the SINR. As a consequence, the present paper investigates the performance of the multihop NOMA systems with the help of FD relaying.

The performance of multihop, FD, and NOMA systems was studied widely in [21,22,23,24,25,26,27]. Xu and others proposed three solutions, namely, stochastic algorithm, two-stage greedy randomized adaptive search, and two-stage stochastic sample to maximize sum-rate by jointly optimizing the channel and power allocation [21]. The outage probability (OP) and ergodic capacity of the NOMA systems combined with full-duplex relaying were derived in [22]. It, however, does not take into account the imperfect successive interference cancellation (SIC) as well as the impact of the near-field path-loss in full-duplex relaying. The closed-form expression of the coverage probability (Pcov) of multiple-input multiple-output (MIMO) cellular networks was given in [23]. Authors in [24] studied the NOMA-enabled unmanned aerial vehicle (UAV) systems under the impact of hardware impairment. More precisely, they derive the OP in the effective computation form. However, they do not consider full-duplex relaying as well as multihop communications that significantly enhance the system spectral efficiency. The combination of NOMA and FD relaying was derived in [26]. To be more specific, the authors derived the OP of the considered networks. They, however, employ the dual-hop relaying and amplify-and-forward (AF) protocol at relay while in the present work, multihop and decode-and-forward (DF) protocol is used. Mujtaba and others in [27] provided a comprehensive discussion about the cooperative power-based NOMA systems in both AF and DF protocols.

The works in [28,29,30,31,32] studied the performance of the full-duplex relaying. In particular, Tan and other authors in [28] addressed the OP and SE of the full-duplex relaying under two relay selection schemes, namely, partial relay selection (PRS) and full relay selection (FRS). The results unveil that the symbol error rate (SER) of the FRS scheme dramatically facilitates. Nevertheless, they do not apply the near-field path-loss that is much more important in FD-enabled communications. The two-way half-duplex relaying with the direct link was studied in [29]. A tractable closed-form expression of the Pcov in Poisson cellular networks was given in [30]. Their outcomes illustrate that the Pcov based on signal-to-interference-plus-noise ratio can be accurately approximated by the proposed definition which is a joint probability of signal-to-interference ratio (SIR) and signal-to-noise ratio (SNR). The statistics of 5G massive MIMO exposure were conducted in [31]. The combination of FD with simultaneous wireless information and power transfer (SWIPT) in wireless sensor networks (WSNs) was investigated in [32]. They, nonetheless, do not use NOMA technique to boost up the SE. The combination of FD relaying SWIPT was studied in [33] over nonidentical Rayleigh fading. They, again, do not apply either NOMA or multihop communications.

The performance of multihop communications was studied extensively in [33,34,35,36,37,38,39]. Alnawafa and other authors in [34] proposed a novel routing algorithm that dramatically improves the lifespan, stability, and throughput of the networks compared with the state-of-the-art. Meanwhile, Thanh and others in [35] derived closed-form expressions many ergodic capacities of the multihop DF systems. On the other hand, the secrecy performance of multihop transmission in cluster networks was addressed in [36]. However, they simply considered multihop networks not combining with other advanced techniques such as NOMA and/or full-duplex relaying. Authors in [37] also investigated the secrecy performance of nonlinear SWIPT systems. They, nonetheless, focus on minimizing the total transmission in downlink and uplink power consumption instead of deriving the OP and system throughput. The Pcov performance of SWIPT-enabled cellular networks was derived in [38]. Unfortunately, they do not consider the near-field path-loss and concentrate on the throughput of the networks. Tin and others in [39] investigated the transmit antenna selection (TAS) and selection combining (SC) of multihop transmission in cognitive WSNs under the impact of hardware impairment.

These above-mentioned works either study each technique separately or combine two techniques instead of considering all three technologies. More importantly, they do not take into account the impact of imperfect SIC which is more important and align with the practical employment of NOMA technique. Regarding the FD relaying, they all skip the influences of near-field path-loss which is not true in FD relaying. As a consequence, different from the above-mentioned works, the present paper, investigates the performance of the combination of these techniques suffering from the imperfection of SIC and self-interference at the relay. More precisely, the main contributions and novelties of the present manuscript are given as follows:

- We take into account the impact of the imperfect interference cancellation (IC) at all receivers. We consider the near-field path-loss at relays to better capture the short transmission distance from the transmit and receive antennae at the relay.
- We take into account the interhop interference and self-interference at all relays due to the full-duplex protocol. It, as a consequence, makes the mathematical framework more complicated compared with half-duplex relaying where the orthogonal transmission between hops is employed.
- We derive closed-form expressions of the OP and potential throughput (PT) of the considered systems.
- We unveil the impact of the total transmit power on the performance of both OP and PT by employing rigorously mathematical frameworks instead of numerical computations.
- We provide remarks to highlight the influence of elements in the OP framework.
- We also derive the mathematical framework of the baseline system to highlight the advantage of the proposed system.
- We supply numerical results via the Monte Carlo method to verify the accuracy of the derived mathematical framework.

The remainder of this paper is organized as follows: The system model is given in Section 2. The derivation of key performance metrics, e.g., the OP, and the PT, is provided in Section 3. Numerical results are presented in Section 4. Section 5 concludes the paper.

## 2. System Model

**Figure 1:**
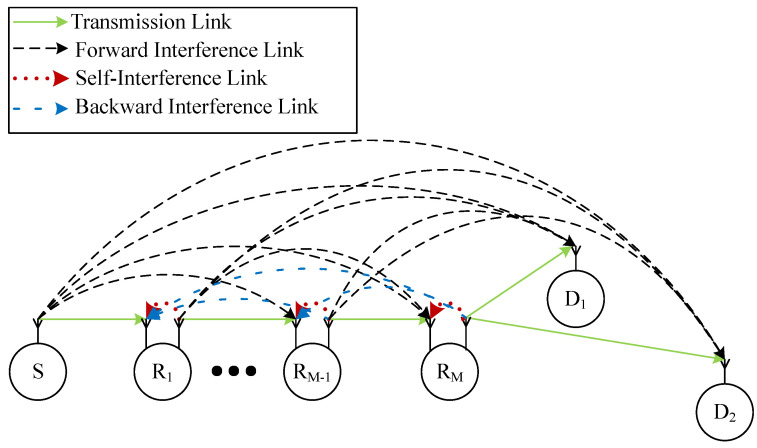
The considered multi-hop FD NOMA networks.

Let us consider a multi-hop NOMA system comprising of a source node denoted by $\left(S_{0}\right)$, M relay nodes denoted by $\left(R_{m}\right)$, $m\in \left\{1,\ldots ,M\right\}$, and 2 destinations denoted by $D_{1}$ and $D_{2}$, as shown in Figure 1. We assume that the source and destinations are equipped with a single antenna while all relay nodes are equipped with two antennae (The considered networks can be applied in several IoT networks, such as Industrial IoT networks [40] and Healthcare IoT systems [41], etc.). As a result, all relays are operated in the full-duplex mode while others are operated in the half-duplex mode. Additionally, thanks to the FD communications, the whole transmission solely takes place in one time slot. Relays employ decode-and-forward protocol instead of amplify-and-forward thanks to its higher performance [42].

### 2.1. Channel Modelling

In the present work, all transmissions are subjected to both small-scale fading and path-loss.

#### 2.1.1. Small-Scale fading

Let us denote $h_{u,v}$ as the channel coefficient from node $u\in \left\{\left(S_{0}\right),\left(R_{1}\right),\ldots ,\left(R_{M}\right)\right\}$ to node $v\in \left\{\left(R_{1}\right),\ldots ,\left(R_{M}\right),D_{1},D_{2}\right\}$ and is followed by a complex Gaussian distribution with zero mean and $\omega_{u,v}$ variance. In the present work, the pilot-based channel estimation is employed to estimate the channel state information (CSI). Particularly, for each hop, a predefined pilot sequence is periodically sent by the transmitter to the receiver to estimate the CSI. The receiver is then sent back this information to the transmitter via a high-accuracy feedback channel. By using this channel estimation method, the multihop communications is decoupled into several conventional single-hop communications [43]. Moreover, we consider the block flat fading that the channel coefficient remains constant for the whole time slot and changes independently between time slots. As a result, the CSI at the transmitters is assumed to be perfect.

#### 2.1.2. Path-Loss

In the present paper, we take into account both near-field and far-field path-loss rather than solely far-field path-loss like works in the literature. The rationale behind such an application is that in FD relaying, the transmission distance between the transmit and receive antennae is probably smaller than the Rayleigh distance (RD), thus the received power at the receive antenna is dominated by the near-field propagation.

##### Far-Field Path-Loss

The far-field path-loss between node $u\in \left\{\left(S_{0}\right),\left(R_{1}\right),\ldots ,\left(R_{M}\right)\right\}$ and node

$v\in \left\{\left(R_{1}\right),\ldots ,\left(R_{M}\right),D_{1},D_{2}\right\}$ denoted by $\varsigma_{u,v}^{F}$ is formulated as follows [44,45]

(1) $$\begin{array}{c} \varsigma_{u,v}^{F}=\frac{4}{G_{T}G_{R}}K_{0}^{2}{\left(d_{u,v}\right)}^{\eta }, \end{array}$$

where $K_{0}=\frac{2\pi }{\lambda }$ and $\lambda =\frac{c}{f_{c}}$ are the wave number and wavelength, $f_{c}$ (in [Hz]) is the carrier frequency, and $c=3\times 10^{8}$ [m/s] is the speed of light, $G_{T}$, $G_{R}$ are the transmit and receive antenna gain, and η is the path-loss exponent. The main notations and mathematical symbols used in the paper is provided in Table 1.

##### Near-Field Path-Loss

Considering the self-interference channel at the relay, it is obvious that both transmit and receive antennae colocate at the same hardware. As a result, the near-field path-loss is necessary to accurately model the propagation of the electromagnetic wave. In fact, in near-field communications, the behavior of the electric and magnetic fields is dissimilar, thus it requires different link equations for each type of antenna. For the electric antenna such as a dipole, the near-field path-loss is formulated as follows [46]:

(2) $$\begin{array}{c} \varsigma_{m}^{N}=\frac{4}{G_{T}G_{R}}{\left(\frac{1}{K_{0}^{2}d_{m}^{2}}-\frac{1}{K_{0}^{4}d_{m}^{4}}+\frac{1}{K_{0}^{6}d_{m}^{6}}\right)}^{-1}. \end{array}$$

The transition from near-field to far-field path-loss is identified via the Rayleigh distance which is given below [47].

(3) $$\begin{array}{c} d_{RD}=\frac{2L^{2}}{\lambda }, \end{array}$$

where *L* is the maximum size of the receiving antenna. Particularly, if $d_{m}>d_{RD}$, (1) is employed to compute the path-loss, otherwise, (2) is used.

### 2.2. Signal-to-Interference-Plus-Noise Ratios (SINRs)

The received signals at the $\left(R_{m}\right)$th relay node with $m\in \left\{1,\ldots ,M\right\}$ are formulated as follows:

(4) $$\begin{array}{cc} y_{\left(R_{m}\right)}= & h_{m-1,m}{\left(\varsigma_{m-1,m}^{F}\right)}^{-1/2}\left(\sqrt{\alpha_{1}P_{m-1}}x_{1}+\sqrt{\alpha_{2}P_{m-1}}x_{2}\right)+\sqrt{P_{m}}h_{m,m}r_{m}{\left(\varsigma_{m}^{N}\right)}^{-1/2}\\ \; & +\sum_{t=0}^{m-2}h_{t,m}{\left(\varsigma_{t,m}^{F}\right)}^{-1/2}\left(\sqrt{\alpha_{1}P_{t}}{\tilde{x}}_{1,t}+\sqrt{\alpha_{2}P_{t}}{\tilde{x}}_{2,t}\right)\\ \; & +\sum_{z=m+1}^{M-1}h_{z,m}{\left(\varsigma_{z,m}^{F}\right)}^{-1/2}\left(\sqrt{\alpha_{1}P_{z}}{\tilde{\tilde{x}}}_{1,z}+\sqrt{\alpha_{2}P_{z}}{\tilde{\tilde{x}}}_{2,z}\right)+n_{m}, \end{array}$$

where $n_{m}$ is the additive white Gaussian noise (AWGN) at the $\left(R_{m}\right)$th relay; $\alpha_{1}$ and $\alpha_{2}$ with $\alpha_{1}+\alpha_{2}=1$, $\alpha_{2}>\alpha_{1}$, are the coefficients of power allocated for $D_{1}$ and $D_{2}$, respectively. $P_{m}$ is the transmit power of the $\left(R_{m}\right)$th relay and $P_{0}$ is the transmit power of source node. Here, we consider the equal power allocation, thus, $P_{m}=P_{0}=P=P_{\mathrm{tot}}/\left(M+1\right),\forall m$; $P_{\mathrm{tot}}$ is the total transmit power of the whole network. $x_{a}$, $a\in \left\{1,2\right\}$, is the signal of interest of $D_{1}$ and $D_{2}$ transmitted by the $\left(R_{m-1}\right)$th relay or source nodes; ${\tilde{\tilde{x}}}_{a,z}$ is the transmitted signals for $D_{a}$ from node $\left(R_{z}\right)$ to node $\left(R_{M}\right)$; and ${\tilde{x}}_{a,t}$ is the novel signals for $D_{1}$ and $D_{2}$ from predecessor nodes, i.e., $\left(S_{0}\right)\to \left(R_{m-1}\right)$. We assume that $E\left\{{\left|x_{a}\right|}^{2}\right\}=E\left\{{\left|{\tilde{x}}_{a,t}\right|}^{2}\right\}=E\left\{{\left|{\tilde{\tilde{x}}}_{a,z}\right|}^{2}\right\}=1,\forall z,a,t$. $r_{m}$ is the residual signals for $D_{a}$, $a\in \left\{1,2\right\}$, at the $\left(R_{m}\right)$th relay after both active and passive cancellation (Passive cancellation can be done by appropriately placing the transmit and receive antenna, i.e., at the two sides of the devices while active cancellation can be employed by using analog and digital SIC circuits [18]) and $E\left\{{\left|r_{m}\right|}^{2}\right\}=\tau_{m}=\tau ,\forall m$.

#### 2.2.1. Perfect Interference Cancellation (PIC)

In this section, we consider the scenario where the successive interference cancellation at relays and destinations is perfect. Additionally, the interference from the successor relays to its processors is canceled out too. The rationale behind this assumption is that since node $\left(R_{m}\right)$ has successfully decoded $x_{1}$ and $x_{2}$ thus it is able to subtract the interference signals from its processors. Hence, the received signals in (4) are then reformulated as follows:

(5) $$\begin{array}{cc} y_{R_{m}}^{P}= & h_{m-1,m}{\left(\varsigma_{m-1,m}^{F}\right)}^{-1/2}\left(\sqrt{\alpha_{1}P_{m-1}}x_{1}+\sqrt{\alpha_{2}P_{m-1}}x_{2}\right)+\sqrt{P_{m}}h_{m,m}r_{m}{\left(\varsigma_{m}^{N}\right)}^{-1/2}\\ \; & +\sum_{t=0}^{m-2}h_{t,m}{\left(\varsigma_{t,m}^{F}\right)}^{-1/2}\left(\sqrt{\alpha_{1}P_{t}}{\tilde{x}}_{1,t}+\sqrt{\alpha_{2}P_{t}}{\tilde{x}}_{2,t}\right)+n_{m}. \end{array}$$

The instantaneous SINR at the (m)th relay to decode $x_{a}$ denoted by $\gamma_{\left(R_{m}\right)}^{x_{a},P}$, $a\in \left\{1,2\right\}$, is then given as

(6) $$\begin{array}{cc} \gamma_{\left(R_{m}\right)}^{x_{1},P}= & \frac{\alpha_{1}{\left|h_{m-1,m}\right|}^{2}\frac{\Phi_{m-1}}{\varsigma_{m-1,m}^{F}}}{\alpha_{2}{\left|h_{m-1,m}\right|}^{2}\frac{\Phi_{m-1}}{\varsigma_{m-1,m}^{F}}+\tau_{m}\frac{\Phi_{m}}{\varsigma_{m}^{N}}{\left|h_{m,m}\right|}^{2}+\sum_{t=0}^{m-2}{\left|h_{t,m}\right|}^{2}\frac{\Phi_{t}}{\varsigma_{t,m}^{F}}+1}\\ \gamma_{\left(R_{m}\right)}^{x_{2},P}= & \frac{\alpha_{2}{\left|h_{m-1,m}\right|}^{2}\frac{\Phi_{m-1}}{\varsigma_{m-1,m}^{F}}}{\tau_{m}\frac{\Phi_{m}}{\varsigma_{m}^{N}}{\left|h_{m,m}\right|}^{2}+\sum_{t=0}^{m-2}{\left|h_{t,m}\right|}^{2}\frac{\Phi_{t}}{\varsigma_{t,m}^{F}}+1}, \end{array}$$

where $\Phi_{m}=P_{m}/\sigma_{m}^{2}=\Phi =P/\sigma^{2},\forall m$, is the average transmit-power-to-noise-ratio; $\sigma^{2}$ is the noise variance of AWGN and is given as $\sigma_{m}^{2}=\sigma^{2}=-174+\mathrm{NF}+10\log\left(\mathrm{Bw}\right),\forall m$; where NF [in dB] is the noise figure; and Bw [in Hz] is the transmission bandwidth. On the other hand, the received signals at node $D_{a}$, $a\in \left\{1,2\right\}$, is computed as:

(7) $$\begin{array}{cc} y_{D_{a}}^{P}=\; & h_{M,D_{a}}{\left(\varsigma_{M,D_{a}}^{F}\right)}^{-1/2}\left(\sqrt{\alpha_{1}P_{M}}x_{1}+\sqrt{\alpha_{2}P_{M}}x_{2}\right)\\ \; & +\sum_{t=0}^{M-1}{\left(\varsigma_{t,D_{a}}^{F}\right)}^{-1/2}h_{t,D_{a}}\left(\sqrt{\alpha_{1}P_{t}}{\tilde{x}}_{1}+\sqrt{\alpha_{2}P_{t}}{\tilde{x}}_{2}\right)+n_{D_{a}}, \end{array}$$

where $h_{M,D_{a}}$ is the channel coefficient from node $\left(R_{M}\right)$ to the *a*th destination. The instantaneous SINR at $D_{1}$ and $D_{2}$ to detect $x_{1}$ and $x_{2}$, is then computed as

(8) $$\begin{array}{cc} \gamma_{D_{1}}^{x_{1},P}= & \frac{\alpha_{1}\frac{\Phi_{M}}{\varsigma_{M,D_{1}}^{F}}{\left|h_{M,D_{1}}\right|}^{2}}{\alpha_{2}\frac{\Phi_{M}}{\varsigma_{M,D_{1}}^{F}}{\left|h_{M,D_{1}}\right|}^{2}+\sum_{t=0}^{M-1}\frac{\Phi_{t}}{\varsigma_{t,D_{1}}^{F}}{\left|h_{t,D_{1}}\right|}^{2}+1},\\ \gamma_{D_{2}}^{x_{2},P}= & \frac{\alpha_{2}\frac{\Phi_{M}}{\varsigma_{M,D_{2}}^{F}}{\left|h_{M,D_{2}}\right|}^{2}}{\sum_{t=0}^{M-1}\frac{\Phi_{t}}{\varsigma_{t,D_{2}}^{F}}{\left|h_{t,D_{2}}\right|}^{2}+1}. \end{array}$$

In the present work, we adopt the decode-and-forward protocol [15], the end-to-end (e2e) SINR to decode $x_{a}$ denoted by $\gamma_{e2e}^{a,P}$, $a\in \left\{1,2\right\}$, is then given as follows:

(9) $$\begin{array}{cc} \gamma_{e2e}^{1,P}= & \min\left(\min_{m=1,2,\ldots ,M}\left(\frac{\alpha_{1}{\left|h_{m-1,m}\right|}^{2}\frac{\Phi_{m-1}}{\varsigma_{m-1,m}^{F}}}{\alpha_{2}{\left|h_{m-1,m}\right|}^{2}\frac{\Phi_{m-1}}{\varsigma_{m-1,m}^{F}}+\tau_{m}\frac{\Phi_{m}}{\varsigma_{m}^{N}}{\left|h_{m,m}\right|}^{2}+\sum_{t=0}^{m-2}{\left|h_{t,m}\right|}^{2}\frac{\Phi_{t}}{\varsigma_{t,m}^{F}}+1}\right),\right.\\ \; & \left.\frac{\alpha_{1}\frac{\Phi_{M}}{\varsigma_{M,D_{1}}^{F}}{\left|h_{M,D_{1}}\right|}^{2}}{\alpha_{2}\frac{\Phi_{M}}{\varsigma_{M,D_{1}}^{F}}{\left|h_{M,D_{1}}\right|}^{2}+\sum_{t=0}^{M-1}\frac{\Phi_{t}}{\varsigma_{t,D_{1}}^{F}}{\left|h_{t,D_{1}}\right|}^{2}+1}\right),\\ \gamma_{e2e}^{2,P}= & \min\left(\min_{m=1,2,\ldots ,M}\left(\frac{\alpha_{2}{\left|h_{m-1,m}\right|}^{2}\frac{\Phi_{m-1}}{\varsigma_{m-1,m}^{F}}}{\tau_{m}\frac{\Phi_{m}}{\varsigma_{m}^{N}}{\left|h_{m,m}\right|}^{2}+\sum_{t=0}^{m-2}{\left|h_{t,m}\right|}^{2}\frac{\Phi_{t}}{\varsigma_{t,m}^{F}}+1}\right),\frac{\alpha_{2}\frac{\Phi_{M}}{\varsigma_{M,D_{2}}^{F}}{\left|h_{M,D_{2}}\right|}^{2}}{\sum_{t=0}^{M-1}\frac{\Phi_{t}}{\varsigma_{t,D_{2}}^{F}}{\left|h_{t,D_{2}}\right|}^{2}+1}\right). \end{array}$$

#### 2.2.2. Imperfect Interference Cancellation (IIC)

Under the imperfect interference cancellation (IIC) scheme, all the assumptions made in Section 2.2.1 are abolished. Particularly, the imperfect SIC is applied at both relays and destinations and the interference from successor relays is taken into consideration too. Hence, from (4), the SINRs at $\left(R_{m}\right)$-th relay under the IIC scheme is rewritten as follows:

(10) $$\begin{array}{cc} \gamma_{\left(R_{m}\right)}^{x_{1},I}= & \frac{\alpha_{1}{\left|h_{m-1,m}\right|}^{2}\frac{\Phi_{m-1}}{\varsigma_{m-1,m}^{F}}}{\alpha_{2}{\left|h_{m-1,m}\right|}^{2}\frac{\Phi_{m-1}}{\varsigma_{m-1,m}^{F}}+\tau_{m}\frac{\Phi_{m}}{\varsigma_{m}^{N}}{\left|h_{m,m}\right|}^{2}+\sum_{t=0,t\neq \left\{m-1,m\right\}}^{M-1}{\left|h_{t,m}\right|}^{2}\frac{\Phi_{t}}{\varsigma_{t,m}^{F}}+1}\\ \gamma_{\left(R_{m}\right)}^{x_{2},I}= & \frac{\alpha_{2}{\left|h_{m-1,m}\right|}^{2}\frac{\Phi_{m-1}}{\varsigma_{m-1,m}^{F}}}{\epsilon_{m}\alpha_{1}{\left|h_{m-1,m}\right|}^{2}\frac{\Phi_{m-1}}{\varsigma_{m-1,m}^{F}}+\tau_{m}\frac{\Phi_{m}}{\varsigma_{m}^{N}}{\left|h_{m,m}\right|}^{2}+\sum_{t=0,t\neq \left\{m-1,m\right\}}^{M-1}{\left|h_{t,m}\right|}^{2}\frac{\Phi_{t}}{\varsigma_{t,m}^{F}}+1}, \end{array}$$

where $\epsilon_{m}\in \left[0,1\right],\forall m$ is the residue of the imperfect SIC. If $\epsilon_{m}=0$ then we return to the perfect cancellation case. Direct inspection (10) and (6), it is certain that the SINR of IIC is consistently smaller than the PIC, i.e., $\gamma_{\left(m\right)}^{x_{a},I}\leq \gamma_{\left(m\right)}^{x_{a},P}$. Although $y_{D_{1}}$ is the same for both PIC and IIC schemes, $y_{D_{2}}$ is not convergent. More precisely, the SINR at $D_{a}$ to detect the $x_{a}$ signals under imperfect IC is written as

(11) $$\begin{array}{cc} \gamma_{D_{1}}^{x_{1},I}= & \frac{\alpha_{1}\frac{\Phi_{M}}{\varsigma_{M,D_{1}}^{F}}{\left|h_{M,D_{1}}\right|}^{2}}{\alpha_{2}\frac{\Phi_{M}}{\varsigma_{M,D_{1}}^{F}}{\left|h_{M,D_{1}}\right|}^{2}+\sum_{t=0}^{M-1}\frac{\Phi_{t}}{\varsigma_{t,D_{1}}^{F}}{\left|h_{t,D_{1}}\right|}^{2}+1},\\ \gamma_{D_{2}}^{x_{2},I}= & \frac{\alpha_{2}\frac{\Phi_{M}}{\varsigma_{M,D_{2}}^{F}}{\left|h_{M,D_{2}}\right|}^{2}}{\epsilon_{D_{2}}\alpha_{1}\frac{\Phi_{M}}{\varsigma_{M,D_{1}}^{F}}{\left|h_{M,D_{1}}\right|}^{2}+\sum_{t=0}^{M-1}\frac{\Phi_{t}}{\varsigma_{t,D_{2}}^{F}}{\left|h_{t,D_{2}}\right|}^{2}+1}. \end{array}$$

Finally the e2e SINRs to decode $x_{a}$ under the IIC scheme is given as

(12) $$\begin{array}{cc} \gamma_{e2e}^{1,I}= & \min\left(\min_{m=1,2,\ldots ,M}\left(\frac{\alpha_{1}{\left|h_{m-1,m}\right|}^{2}\frac{\Phi_{m-1}}{\varsigma_{m-1,m}^{F}}}{\alpha_{2}{\left|h_{m-1,m}\right|}^{2}\frac{\Phi_{m-1}}{\varsigma_{m-1,m}^{F}}+\tau_{m}\frac{\Phi_{m}}{\varsigma_{m}^{N}}{\left|h_{m,m}\right|}^{2}+\sum_{t=0,t\neq \left\{m-1,m\right\}}^{M-1}{\left|h_{t,m}\right|}^{2}\frac{\Phi_{t}}{\varsigma_{t,m}^{F}}+1}\right),\right.\\ \; & \left.\frac{\alpha_{1}\frac{\Phi_{M}}{\varsigma_{M,D_{1}}^{F}}{\left|h_{M,D_{1}}\right|}^{2}}{\alpha_{2}\frac{\Phi_{M}}{\varsigma_{M,D_{1}}^{F}}{\left|h_{M,D_{1}}\right|}^{2}+\sum_{t=0}^{M-1}\frac{\Phi_{t}}{\varsigma_{t,D_{1}}^{F}}{\left|h_{t,D_{1}}\right|}^{2}+1}\right),\\ \gamma_{e2e}^{2,I}= & \min\left(\min_{m=1,2,\ldots ,M}\left(\frac{\alpha_{2}{\left|h_{m-1,m}\right|}^{2}\frac{\Phi_{m-1}}{\varsigma_{m-1,m}^{F}}}{\epsilon_{m}\alpha_{1}{\left|h_{m-1,m}\right|}^{2}\frac{\Phi_{m-1}}{\varsigma_{m-1,m}^{F}}+\tau_{m}\frac{\Phi_{m}}{\varsigma_{m}^{N}}{\left|h_{m,m}\right|}^{2}+\;\;\;\;\sum_{t=0,t\neq \left\{m-1,m\right\}}^{M-1}{\left|h_{t,m}\right|}^{2}\frac{\Phi_{t}}{\varsigma_{t,m}^{F}}+1}\right),\right.\\ \; & \left.\frac{\alpha_{2}\frac{\Phi_{M}}{\varsigma_{M,D_{2}}^{F}}{\left|h_{M,D_{2}}\right|}^{2}}{\epsilon_{D_{2}}\alpha_{1}\frac{\Phi_{M}}{\varsigma_{M,D_{1}}^{F}}{\left|h_{M,D_{1}}\right|}^{2}+\sum_{t=0}^{M-1}\frac{\Phi_{t}}{\varsigma_{t,D_{2}}^{F}}{\left|h_{t,D_{2}}\right|}^{2}+1}\right). \end{array}$$

## 3. Performance Analysis and Trends

In the present work, we address the performance of two vital metrics, namely, the outage probability and the potential throughput. The former calculates the probability that the instantaneous e2e SINR of both destinations is below the predefined threshold, the latter, on the other hand, computes the potential throughput of the whole network. Mathematical speaking, these metrics can be formulated as follows [48]:

(13) $$\begin{array}{cc} {\mathrm{OP}}_{a}^{w}= & \mathrm{Pr}\left\{\log_{2}\left(1+\gamma_{e2e}^{a,w}\right)\leq R_{a}\right\},\;\;\;\;w\in \left\{P,I\right\}\\ \mathrm{PT}= & \sum_{a=1}^{2}\log_{2}\left(1+R_{a}\right)\left(1-OP_{a}\right). \end{array}$$

Here, $\mathrm{Pr}\left\{.\right\}$ is the probability operator, $\log\left(.\right)$ is the logarithm function, and $R_{a}$ is the expected rate of the *a*th destination. To compute the OP and PT, the following Theorem is useful and is given as follows:

**Theorem** **1.** *Considering a set of N independently and non-identically distributed (i.n.i.d.) exponential random variables (RVs) with scale $\delta_{n}$, i.e., $X_{n},n\in \left\{1,\ldots ,N\right\}$, the moment generating function (MGF) of summation of these RVs, i.e., $S=\sum_{n=1}^{N}X_{n}$ denoted by $M_{S}\left(s\right)$ are then given by [49]*

(14) $$\begin{array}{c} M_{S}\left(s\right)=\prod_{i=1}^{N}\frac{1}{1+s\delta_{i}}. \end{array}$$

**Proof.**  The proof is given in Appendix A. □

Having obtained the MGF of the sum of M i.n.i.d. exponential RVs, the OP of $D_{a}$, $a\in \left\{1,2\right\}$ under both PIC and IIC schemes are given in Theorems 2 and 3 as follows:

**Theorem** **2.** *Let us represent the e2e SINR to decode x${}_{1}$ and x${}_{2}$ under the PIC scheme as $\gamma_{e2e}^{1,P}=\min_{i\in \left\{1,\ldots ,M,D_{1}\right\}}\left\{W_{i}=\frac{a_{i}X_{i}}{b_{i}X_{i}+\sum_{t=1}^{N_{i}}c_{t,i}Y_{t,i}+d_{i}}\right\}$ and $\gamma_{e2e}^{2,P}=\min_{i\in \left\{1,\ldots ,M,D_{2}\right\}}\left\{Z_{i}=\frac{a_{i}X_{i}}{\sum_{t=1}^{N_{i}}c_{t,i}Y_{t,i}+d_{i}}\right\}$,* *where $a_{i},b_{i},c_{t,i},d_{i}$ are real numbers, $N_{i}$ is a positive integer, $X_{i}$ and $Y_{t,i}$ are independently exponential RVs with distinct parameters, and $W_{i},Z_{i},\forall i$ are independent of each other. The OP of the ath destination denoted by ${\mathrm{OP}}_{a}^{P}$, $a\in \left\{1,2\right\}$, is represented in an unified expression and is given by*

(15) $$\begin{array}{cc} {\mathrm{OP}}_{a}^{P}= & 1-H\left(\Delta_{a}\right)\left[\exp\left(-\frac{\lambda_{a}\varsigma_{M,D_{a}}^{F}}{\Phi \omega_{M,D_{a}}}\right)\prod_{l=1}^{M}{\left(1+\lambda_{a}\frac{\varsigma_{M,D_{a}}^{F}\omega_{l-1,D_{a}}}{\varsigma_{l-1,D_{a}}^{F}\omega_{M,D_{a}}}\right)}^{-1}\right]\left[\prod_{z=1}^{M}\exp\left(-f_{a}\left(z\right)\right)\right]\\ \; & \times \left[\prod_{i=1}^{M}{\left(1+\tau_{i}\omega_{i,i}\frac{\Phi }{\varsigma_{i}^{N}}\right)}^{-1}\right]\left[\prod_{m=2}^{M}\prod_{o=2}^{m}{\left(1+f_{a}\left(m\right)\frac{\Phi }{\varsigma_{o-2,m}^{F}}\omega_{o-2,m}\right)}^{-1}\right], \end{array}$$

*where $\lambda_{a}=\frac{\gamma_{\mathrm{th}}^{a}}{\Delta_{a}}$, $\Delta_{1}=\alpha_{1}-\alpha_{2}\gamma_{\mathrm{th}}^{1}$ and $\Delta_{2}=\alpha_{2}$, $f_{a}\left(x\right)=\frac{\lambda_{a}\varsigma_{x-1,x}^{F}}{\left(\Phi_{x-1}\omega_{x-1,x}\right)}$, $\gamma_{\mathrm{th}}^{a}=2^{R_{a}}-1$, $\exp\left(.\right)$ is the exponential function and $H\left(.\right)$ is the Heaviside function.*

**Proof.**  The proof is available at Appendix B. □

**Remark** **1.** *Direct inspection (15) we observe that the impact of the interference at $D_{a}$ and relays on OP are given by the terms $\prod_{l=1}^{M}{\left(1+\lambda_{a}\frac{\varsigma_{M,D_{a}}^{F}\omega_{l-1,D_{a}}}{\varsigma_{l-1,D_{a}}^{F}\omega_{M,D_{a}}}\right)}^{-1}$ and $\prod_{m=2}^{M}\prod_{o=2}^{m}{\left(1+f_{a}\left(m\right)\frac{\Phi }{\varsigma_{o-2,m}^{F}}\omega_{o-2,m}\right)}^{-1}$ while the influence of the self-interference at relays is given by $\prod_{i=1}^{M}{\left(1+\tau_{i}\omega_{i,i}\frac{\Phi }{\varsigma_{i}^{N}}\right)}^{-1}$.*

**Theorem** **3.** *Let us represent the e2e SINR to decode x${}_{a}$, $a\in \left\{1,2\right\}$, under the IIC scheme as* *$\gamma_{e2e}^{a,P}=\min_{i\in \left\{1,\ldots ,M,D_{a}\right\}}\left\{U_{i}=\frac{a_{i}X_{i}}{b_{i}X_{i}+\sum_{t=1}^{N_{i}}c_{t,i}Y_{t,i}+d_{i}}\right\}$ where $a_{i},b_{i},c_{t,i},d_{i}$ are real numbers, $N_{i}$ is a positive integer, $X_{i}$ and $Y_{t,i}$ are independently exponential RVs with distinct parameters, and $U_{i},\forall i$ are independent of each other. The OP of the ath destination denoted by ${\mathrm{OP}}_{a}^{I}$, $a\in \left\{1,2\right\}$, is given by*

(16) $$\begin{array}{cc} {\mathrm{OP}}_{1}^{I}= & 1-H\left(\Delta_{1}\right)\exp\left(-\frac{\lambda_{1}\varsigma_{M,D_{1}}^{F}}{\Phi \omega_{M,D_{1}}}\right)\prod_{m=2}^{M-1}\exp\left(-f_{1}\left(m\right)\right){\left(1+f_{1}\left(m\right)\tau_{m}\omega_{m,m}\frac{\Phi_{m}}{\varsigma_{m}^{N}}\right)}^{-1}\\ \; & \times \left[\prod_{z=0,z\neq \left\{m-1,m\right\}}^{M-1}{\left(1+f_{1}\left(m\right)\frac{\Phi }{\varsigma_{z,m}^{F}}\omega_{z,m}\right)}^{-1}\right]\prod_{l=1}^{M}{\left(1+\lambda_{1}\frac{\varsigma_{M,D1}^{F}\omega_{l-1,D1}}{\varsigma_{l-1,D1}^{F}\omega_{M,D1}}\right)}^{-1}\\ {\mathrm{OP}}_{2}^{I}= & 1-H\left(\Delta_{2}\right)H\left(\Delta_{3}\right)\exp\left(-\frac{\lambda_{2}\varsigma_{M,D_{2}}^{F}}{\Phi \omega_{M,D_{2}}}\right)\prod_{m=1}^{M}\exp\left(-f_{3}\left(m\right)\right){\left(1+f_{3}\left(m\right)\tau_{m}\omega_{m,m}\frac{\Phi_{m}}{\varsigma_{m}^{N}}\right)}^{-1}\\ \; & \times \left[\prod_{z=0,z\neq \left\{m-1,m\right\}}^{M}{\left(1+f_{3}\left(m\right)\frac{\Phi }{\varsigma_{z,m}^{F}}\omega_{z,m}\right)}^{-1}\right]\prod_{l=1}^{M}{\left(1+\lambda_{2}\frac{\varsigma_{M,D_{2}}^{F}\omega_{l-1,D_{2}}}{\varsigma_{l-1,D_{2}}^{F}\omega_{M,D_{2}}}\right)}^{-1}\\ \; & \times {\left(1+\lambda_{2}\epsilon_{D_{2}}\alpha_{1}\frac{\varsigma_{M,D_{2}}^{F}\omega_{M,D_{1}}}{\varsigma_{M,D_{1}}^{F}\omega_{M,D_{2}}}\right)}^{-1}, \end{array}$$

*where $\Delta_{3}=\alpha_{2}-\epsilon \alpha_{1}\gamma_{\mathrm{th}}^{2}$, $\upsilon =\frac{\gamma_{\mathrm{th}}^{2}}{\Delta_{3}}$ and $f_{3}\left(x\right)=\frac{\upsilon \varsigma_{x-1,x}^{F}}{\Phi_{x-1}\omega_{x-1,x}}$.*

**Proof.**  The proof is available at Appendix C. □

The potential throughput of the whole networks is then straightforwardly computed by substituting ${\mathrm{OP}}_{a}^{w}$, $w\in \left\{P,I\right\}$, $a\in \left\{1,2\right\}$, in (15), (16) into (13).

### 3.1. Performance Trends

The behaviors of the considered metrics with respect to a key parameter are revealed in this section. Particularly, the impact of $P_{\mathrm{tot}}$ on the performance of OP and PT is given in the following Proposition.

**Proposition** **1.** *The OP monotonically decreases with respect to the total transmit power while the PT simply increases with this parameter.*

**Proof.**  The proof is available at Appendix D. □

### 3.2. Performance of Baseline System

In this section, we provide the closed-form expression of the single-hop communications from $\left(S_{0}\right)$ to $D_{1}$ and $D_{2}$ without the assistance of relays. The OP of *D*${}_{a}$ under the baseline system denoted by ${\mathrm{OP}}_{a}^{\mathrm{bas}}$ is then computed as follows:

(17) $$\begin{array}{c} {\mathrm{OP}}_{a}^{\mathrm{bas}}=1-\exp\left(-\frac{\sigma^{2}\lambda_{a}}{\left(P_{\mathrm{tot}}\varsigma_{0,D_{a}}^{F}\right)}\right). \end{array}$$

To ensure a fair comparison, the transmit power of the source node is fully allocated and is equal to $P_{\mathrm{tot}}$.

## 4. Simulation Results

In this section, we provide numerical results to verify the accuracy of the proposed mathematical framework as well as to unveil the behaviors of the considered metrics as a function of some key parameters. Unless otherwise stated, the following parameters are adopted which are based on the narrowband IoT (NB-IoT) networks: M=3, Bw = 500 kHz, η=3.75, $f_{c}=2.1$ GHz, NF = 6 dB, $P_{\mathrm{tot}}$ = 30 dBm, $R_{1}=R_{2}=R=0.075$ [bits/s/Hz]; $\alpha_{1}=0.6$; $\alpha_{2}=1-\alpha_{1}=0.4$, $G_{T}=G_{R}=0$ dB, ϵ=0.01, $d_{R,R}$ = 0.5 [m], *L* = 0.3 [m], ω = 1, and τ = −100 dB. The position of source node is at (0,0) [m], the positions of $D_{1}$ and $D_{2}$ are (1200,20) and (1200,−20), respectively. We assume that all relay nodes are located on a straight line and equally separated, their locations are then given as (300,0), (600,0), and (900,0).

**Figure 2:**
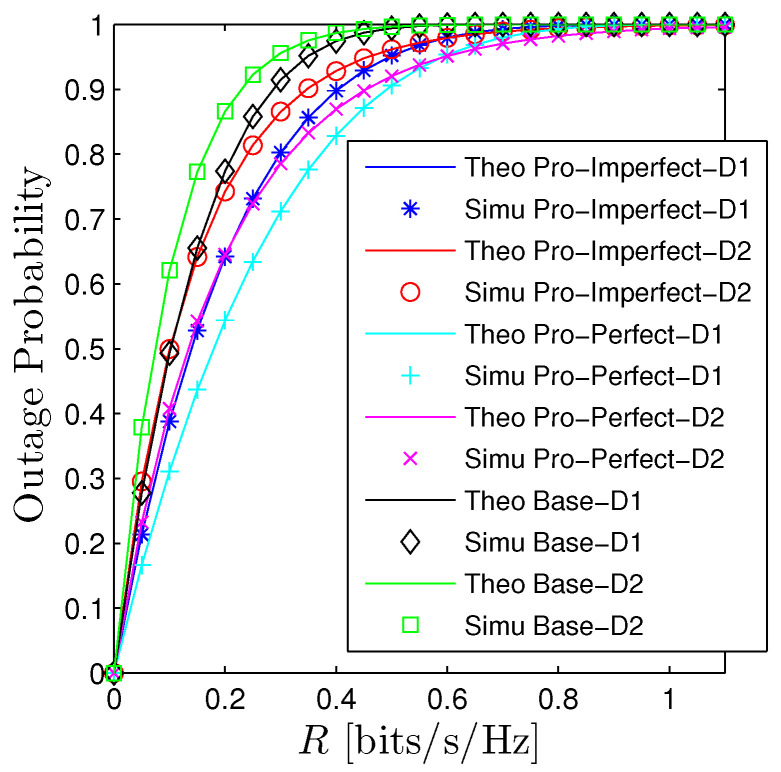
Outage probability vs. *R* under all schemes. Solid lines are plotted by employing (15)–(17) while markers are Monte Carlo simulation.

**Figure 3:**
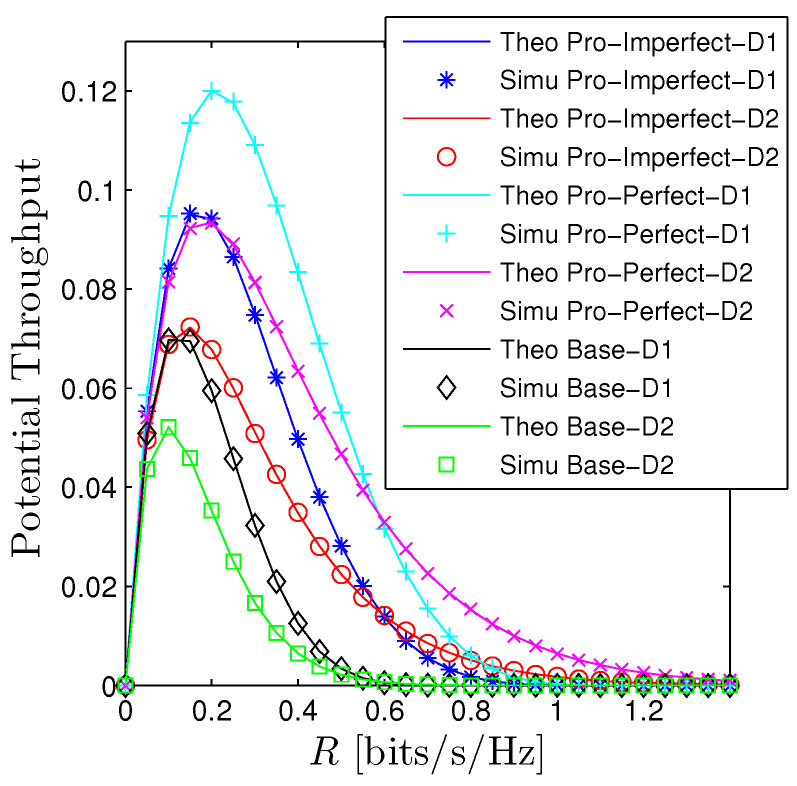
Potential throughput vs. *R* under all schemes. Solid lines are plotted by employing (13) while markers are Monte Carlo simulation.

Figure 2 and Figure 3 illustrates the performance of the OP and PT with respect to the expected rate $R_{1}=R_{2}=R$. We observe that there is a good agreement between the derived mathematical framework and the Monte Carlo simulations for all schemes. The OP of *D*${}_{1}$ under the PIC scheme denoted by “Pro-Perfect-D1” is better than the OP of *D*${}_{2}$ denoted by “Pro-Perfect-D2” when *R* is relatively small. However, when OP is approaching 1, the OP of *D*${}_{2}$ is better. We observe a similar trend for the IIC and baseline system. It is certain that under the IIC scheme, the OP performance will obviously be worse than the PIC. More precisely, the OP of D${}_{a}$, $a\in \left\{1,2\right\}$ under the IIC denoted by “Pro-Imperfect-D${}_{a}$” is higher the curve “Pro-Perfect-D${}_{a}$” approximately 0.1 when *R* is around 0.3. Nonetheless, the OP of the IIC scheme is still better than the baseline system (denoted by “Base-D1” and “Base-D2”). Particularly, the OP under the baseline system already reaches 1 when *R* is around 0.3 while the OP of the PIC only reaches 1 when *R* is slightly below 1. Regarding the PT, we also observe a big gap between the proposed systems and the baseline one. Moreover, the PT is a unimodal function concerning the expected rate. It can be straightforwardly explained that when *R* is fairly small the PT is dominated by the term $\log_{2}\left(1+R\right)$ so PT is increasing and when *R* is getting bigger the impact of OP becomes the major player thus PT decreases and approaches zero when R≫1.

**Figure 4:**
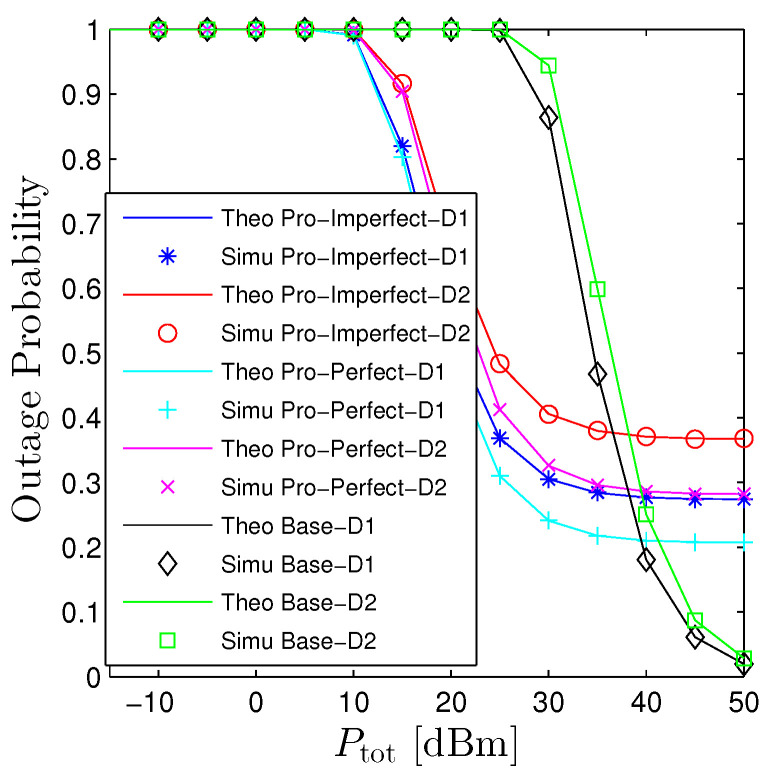
Outage probability vs. $P_{\mathrm{tot}}$ under all schemes. Solid lines are plotted by employing (15)–(17) while markers are Monte Carlo simulation.

**Figure 5:**
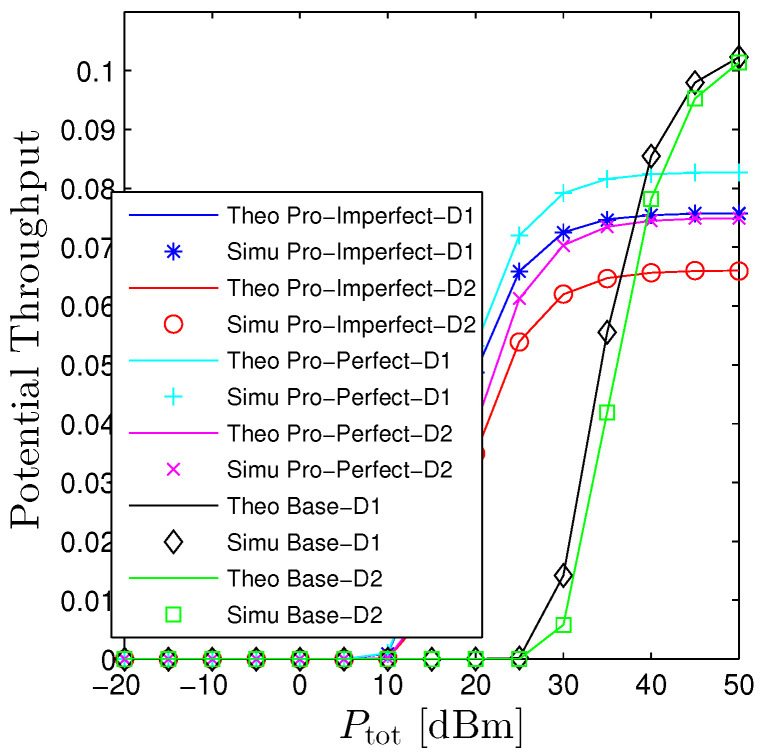
Potential throughput vs. $P_{\mathrm{tot}}$ under all schemes. Solid lines are plotted by employing (13) while markers are Monte Carlo simulation.

Figure 4 and Figure 5 investigates the influences of $P_{\mathrm{tot}}$ on the performance of OP and PT. This figure confirms again that the proposed mathematical framework is consistently aligned with computer-based simulation results. Furthermore, it also verifies the statements in Proposition 1 that increasing $P_{\mathrm{tot}}$ monotonically declines the OP and raises the PT. It is interesting to point out that the proposed system (even under the PIC scheme) does not substantially outperform its counterpart. In fact, the proposed network provides better performance when the transmit power is small, moderate, and worse when $P_{\mathrm{tot}}\gg 1$. The rationale behind this phenomenon is that when the system is under a high transmit power regime, both destinations are able to successfully decode signals without the help of relays. Nonetheless, under the proposed system, there always exists self-interference at relays and the interinterference between hops thus degrading the system. On the other hand, when the transmit power is either small or moderate, the OP of the baseline system underperforms the proposed one owing to the long transmission distance. Figure 5 shows the performance of PT concerning $P_{\mathrm{tot}}$. We see that it has a reverse trend compared with the OP that monotonically increases with $P_{\mathrm{tot}}$. It is clear that in order to get the nonzero PT, the baseline system requires $P_{\mathrm{tot}}$ is greater than 25 dBm while the proposed system solely demands the $P_{\mathrm{tot}}$ is greater than 10 dBm for the worst scenario (all-hop interference).

**Figure 6:**
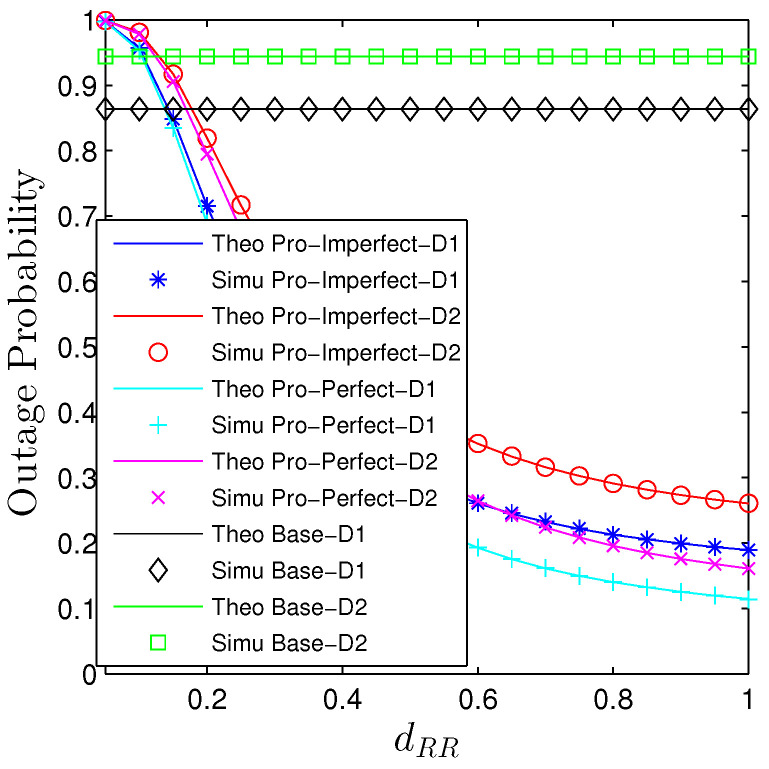
Outage probability vs. $d_{m,m}$ under all schemes. Solid lines are plotted by employing (15)–(17) while markers are Monte Carlo simulation.

**Figure 7:**
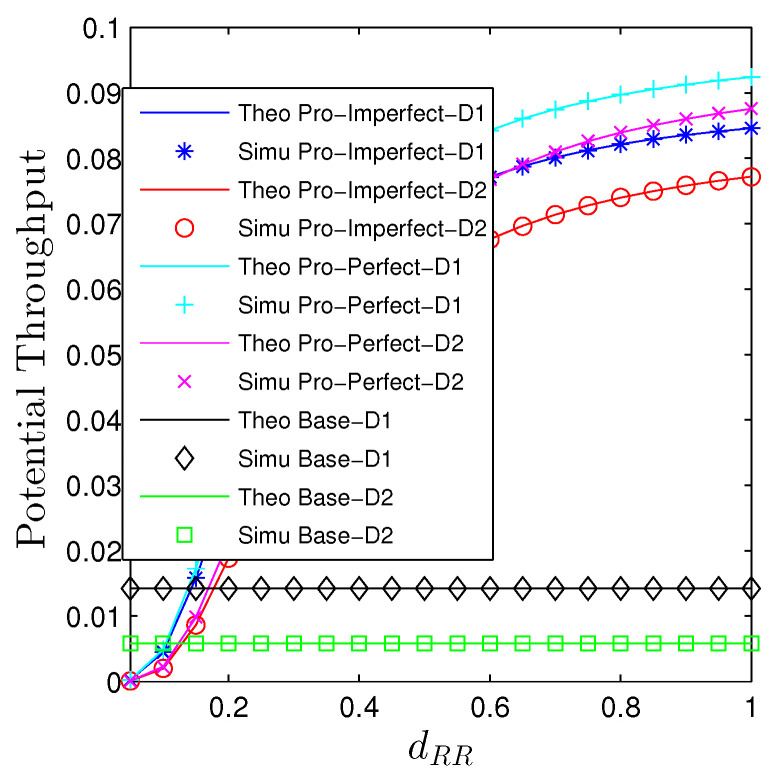
Potential throughput vs. $d_{m,m}$ under all schemes. Solid lines are plotted by employing (13) while markers are Monte Carlo simulation.

The influences of the near-field path-loss are conducted in Figure 6 and Figure 7. Particularly, we see that increasing the transmit distance between the transmit and receive antennae at the relay will monotonically decrease the OP and increase the PT. It can be explained straightforwardly from (2) that increasing $d_{RR}$, the impact of two terms that are the power of 4 and 6 of the transmission distance approach zeros faster than the power of 2. Hence, the near-field path-loss gradually becomes the large-scale path-loss. It is expected that the baseline curves are constant with the changes in the self-interference transmission distance. Under the current setup, the proposed system substantially outperforms the baseline one unless $d_{RR}<0.2$.

**Figure 8:**
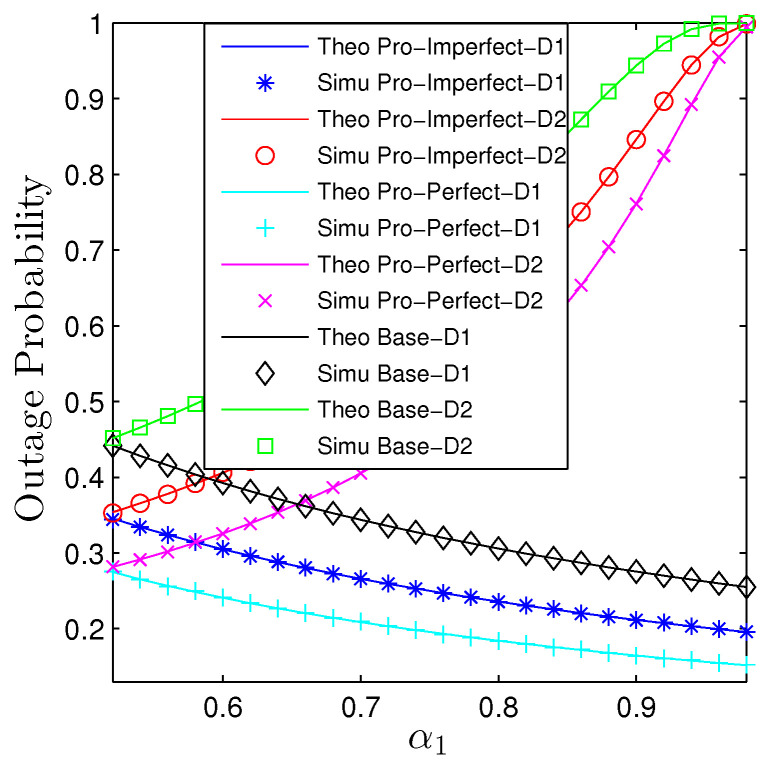
Outage probability vs. $\alpha_{1}$ under all schemes. Solid lines are plotted by employing (15)–(17) while markers are Monte Carlo simulation.

**Figure 9:**
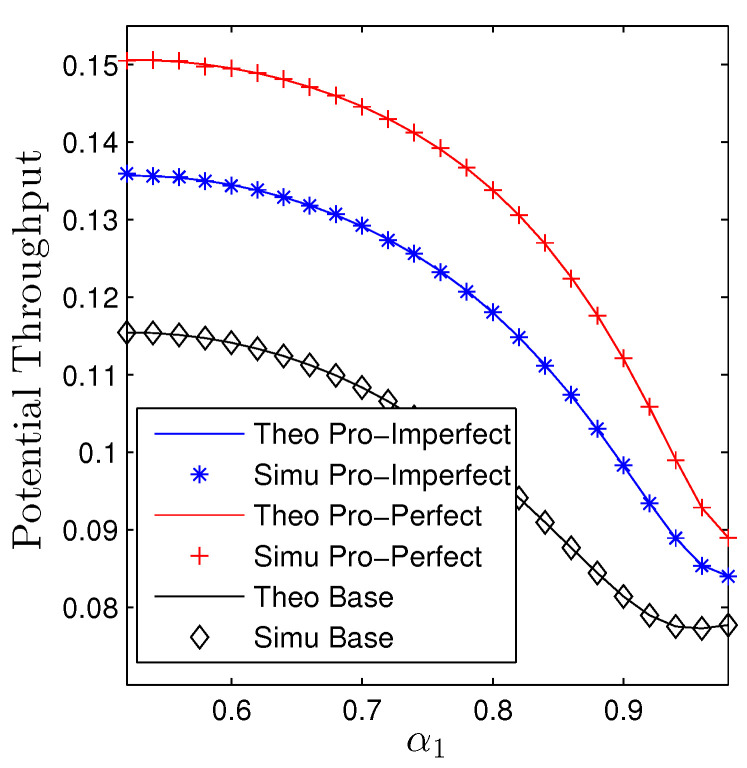
Potential throughput vs. $\alpha_{1}$ under all schemes. Solid lines are plotted by employing (13) while markers are Monte Carlo simulation.

The impact of $\alpha_{1}$ on the performance of both metrics is given in Figure 8 and Figure 9. It is certain that increasing $\alpha_{1}$ is beneficial for D${}_{1}$, it, however, will be harmful to D${}_{2}$ regardless of the utilized schemes and IC conditions. Nevertheless, the increase and decrease paces are different. We observe that the OP${}_{2}$ dramatically increases while the decrease of OP${}_{1}$ is moderate. Similar trends are observed for the baseline system. Figure 9 illustrates the influences of $\alpha_{1}$ on the performance of potential throughput of the whole networks. Specifically, scaling up $\alpha_{1}$ degrades the performance of the considered networks as well as the baseline ones. Additionally, we observe a quite big gap between the proposed networks and the nonrelaying systems regardless of the conditions of the interference cancellation.

**Figure 10:**
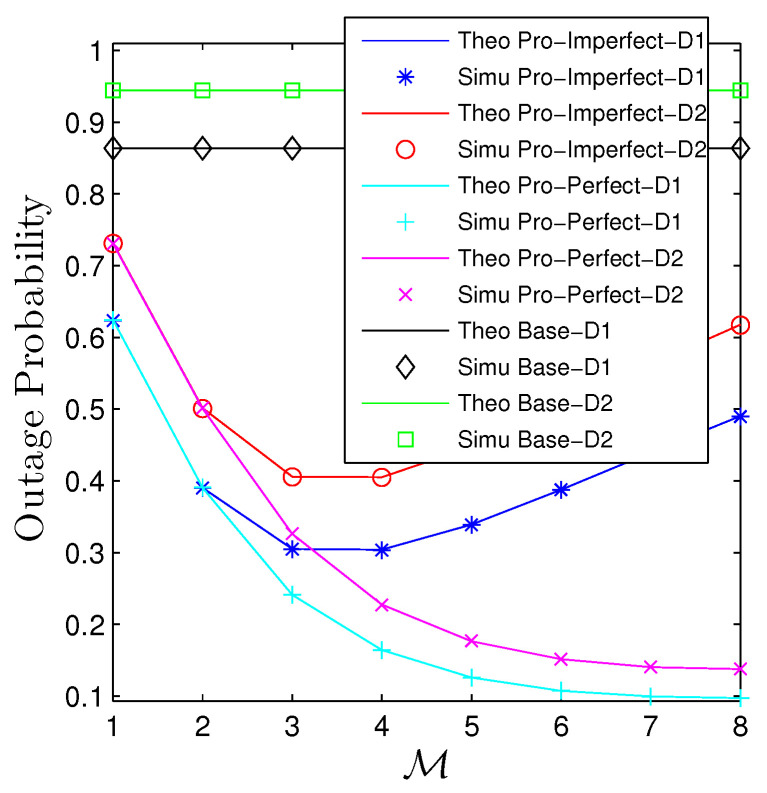
Outage probability vs. M under all schemes. Solid lines are plotted by employing (15)–(17) while markers are Monte Carlo simulation.

**Figure 11:**
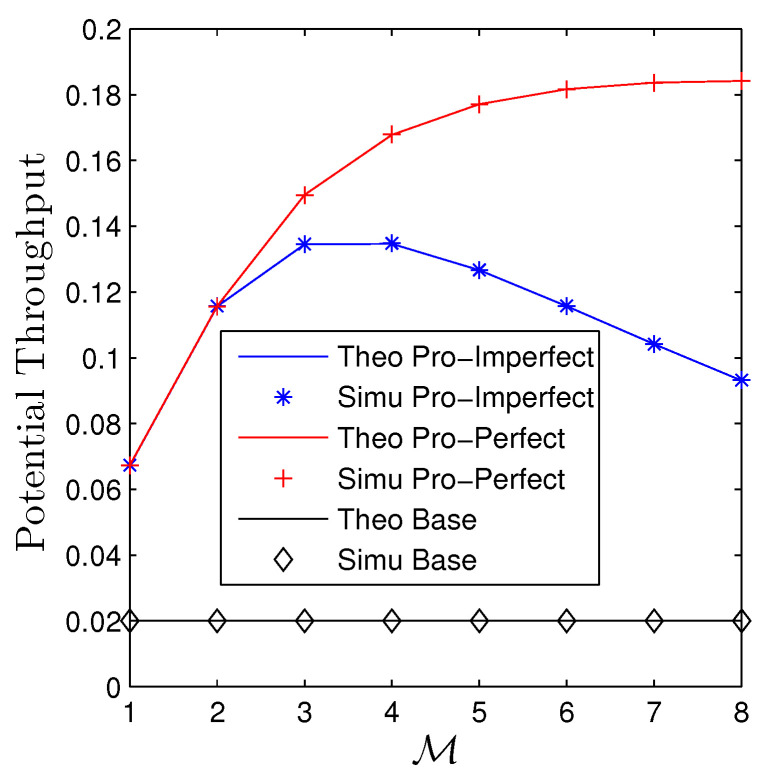
Potential throughput vs. M under all schemes. Solid lines are plotted by employing (13) while markers are Monte Carlo simulation.

Figure 10 and Figure 11 addresses the influences of the number of relays on the performance of two metrics. It is evident that the baseline curves are constant with M. Regarding the considered networks, we observe that the OP quickly decreases when M is small, i.e., M≤3 for both scenarios. It then slowly decreases followed by slight increases when M>8 for the PIC. Regarding the IIC, the OP remains constant for M=3 and M=4. It then dramatically increases when M≥5. The behavior of PT, contrarily, experiences a reverse trend with OP that it starts increasing until the peak then steadily declines, and the PIC, of course, achieves higher throughput than IIC.

**Figure 12:**
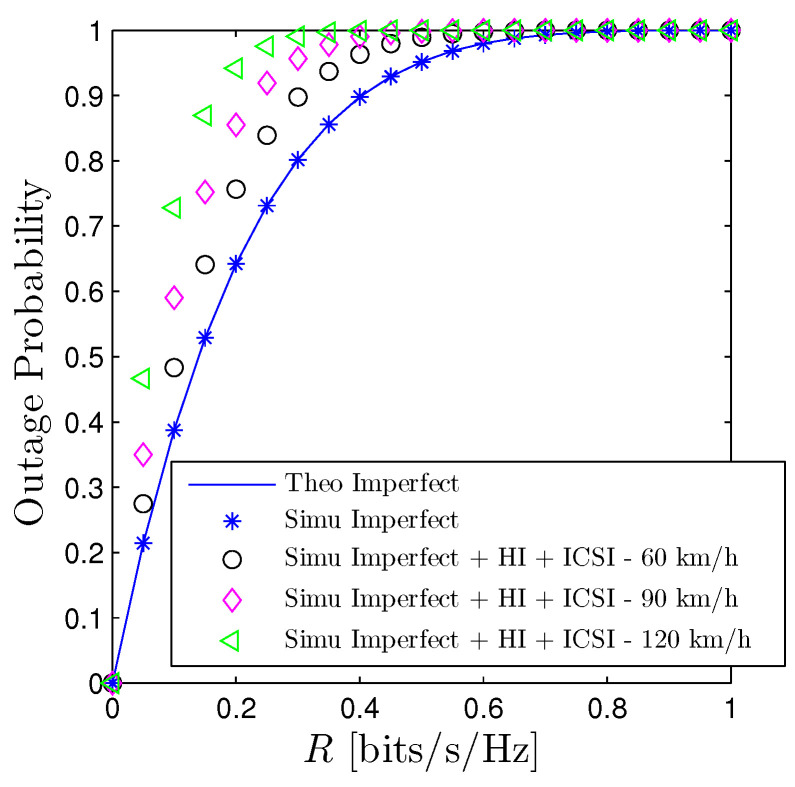
Outage probability vs. *R* under the impact of imperfect interference cancellation, hardware impairment (HI), and imperfect channel state information (ICSI). Solid lines are plotted by employing (16) while markers are Monte Carlo simulation.

Figure 12 depicts the performance of the OP of D${}_{1}$ under the impact of imperfect interference cancellation, hardware impairment, and imperfect channel estimation with respect to *R*. More precisely, we adopt the imperfect channel state information (ICSI) as in [50] that the channel coefficient of a generic link between transmitter *u* and receiver *v* is modelled as ${\tilde{h}}_{u,v}=\rho h_{u,v}+\sqrt{1-\rho^{2}}w_{u,v}$, where ${\tilde{h}}_{u,v}$ is the estimated version of $h_{u,v}$ and $w_{u,v}$ is a complex Gaussian RV with zero mean and the same variance as $h_{u,v}$. Here $\rho \in \left[0,1\right]$ is the correlation coefficient and is computed by $J_{0}\left(2\pi f_{D}\psi \right)$, where $J_{0}\left(.\right)$ is the zeroth-order Bessel function of the first kind, $f_{D}=v_{r}\cos\left(\nu \right)f_{c}/c$ is the Doppler shift, ν is the angle between wave propagation and motion direction, $v_{r}$ is the relative velocity of receiver, and ψ is the feedback delay. In Figure 12, we select $v_{r}=\left\{60,90,120\right\}$ km/h, ν = 60${}^{\circ }$ and ψ=0.5 ms [51]. The curves denoted by “Simu Imperfect + HI + ICSI” are plotted by assuming that all imperfect factors, i.e., interference cancellation, hardware impairment (HI), and ICSI are imposed on the system while the curves denoted by “Theo Imperfect” are the IIC scheme. Regarding the hardware impairment, the HI model in [52] is taken into account where the transmit signals are impaired by an additive noise which follows a circularly symmetric complex Gaussian distribution with zero-mean and variance Pϖ, $\varpi \in \left[0,1\right]$. In this figure, we choose ϖ=0.075 as like in [52]. From Figure 12, it is expected that OP under the influences of imperfect interference cancellation, hardware impairment, and imperfect channel state information have similar behaviors as case only imperfect interference cancellation but got worse performance. One of the most effective solutions to overcome such harmful effects is to increase the transmit power as shown in Figure 4. We see that the impact of HI is minor compared with the imperfect CSI. Furthermore, the higher the ρ, the better the OP.

**Figure 13:**
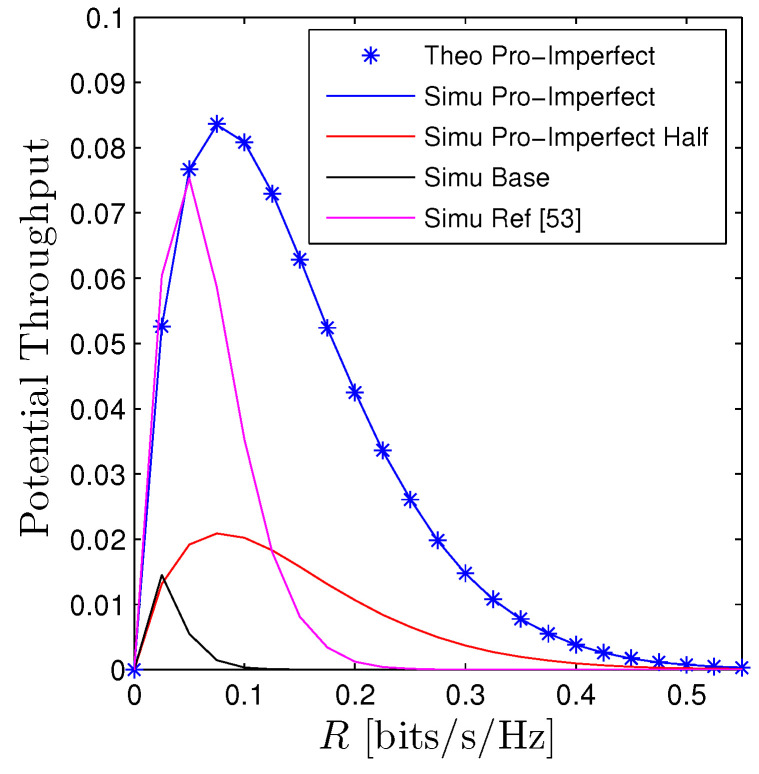
Potential throughput vs. *R* with various schemes, full-duplex, half-duplex, state-of-the-art [53], and baseline system. Markers are plotted by employing (13) while solid lines are Monte Carlo simulation.

Figure 13 unveils the performance of the PT as a function of *R* under different schemes, i.e., full-duplex, half-duplex, state-of-the-art [53], and baseline systems. More precisely, for the line denoted by “Simu Pro-Imperfect Half”, all relays operate at half-duplex protocol where relays can only either transmit or receive signals at each time slot thus, the whole transmission takes place in M+1 time slots. To make a fair comparison, all relays still employ two antennae and both transmit and receive diversity schemes are deployed such as maximal ratio transmission (MRT) and maximal ratio combining (MRC) to maximize its performance. Additionally, the self-interference and interhop interference are also removed. Regarding the line denoted by “Simu Ref. [53]”, we consider a dual-hop FD NOMA networks with a help of a single DF relay in [53]. In their work, both source and destinations are equipped with multiple antennae thus, both MRT and MRC are used to maximize the system performance. The remaining curves are given by (13), (16) and (17). It is no doubt that our proposed scheme achieves the highest PT followed by the scheme in [53], half-duplex and the lowest one is the baseline system. Although the half-duplex protocol enjoys free interhop interference and self-interference at the relay, requiring several time slots to forward information significantly scales down its throughput. As for the scheme in [53], it benefits from a favorable channel gain thanks to transmit and receive diversity techniques, it, however, suffers from a long transmission distance. As a result, the performance still underperforms the proposed scheme.

## 5. Conclusions

The performance of the multi-hop FD NOMA systems was addressed in this paper. More precisely, we derived the OP and PT in the closed-form expressions for two cases, perfect and imperfect IC. Moreover, we also derived the behaviors of these metrics with respect to an important parameter, namely, the total transmit power. The results unveiled that OP and PT have a contrary trend. We also identified that when the transmit power goes without bound, the baseline system outperforms the proposed one. The ongoing extension of the current work is to take into account the impact of the imperfect hardware and imperfect CSI by deriving mathematical frameworks in order to comprehensively address the performance of the considered networks.

## Figures and Tables

**Table 1:** Main notations and mathematical symbols.

Symbol	Definition
$E\left\{.\right\}$, $\mathrm{Pr}\left(.\right)$	Expectation and probability operators
$h_{u,v}$	Channel coefficient between transmitter *u* and receiver *v*
$\varsigma_{u,v}^{F}$	Far-field path-loss between transmitter *u* and receiver *v*
$\varsigma_{m}^{N}$	Near-field path-loss between of the $R_{m}$ relay
$K_{0}$, *c*	Path-loss constant, speed of light
*v*, $f_{c}$, η	Wavelength, carrier frequency, path-loss exponent
$d_{u,v}$	Transmission distance from node *u* to node *v*
$P_{\mathrm{tot}}$	Total transmit power of the whole networks
$P_{m}$, $P_{0}$	Transmit power of the $R_{m}$ relay and source node
$\alpha_{1}$, $\alpha_{2}$	Coefficients of power allocation for $D_{1}$ and $D_{2}$
*L*, M	Maximum size of the received antenna & number of relays
$d_{RD}$, $G_{T}$, $G_{R}$	Rayleigh distance, transmit and receive antennae gain
$R_{a}$, $a\in \left\{1,2\right\}$	Targeted rate of $D_{a}$ destination
$\varepsilon_{v}$	Residue of the imperfect SIC at *v* receiver
$r_{m}$	Residue of the self-interference cancellation at $R_{m}$ relay
$x_{a}$, ${\tilde{x}}_{a}$, ${\tilde{\tilde{x}}}_{a}$	Intended signals for $D_{a}$ sent by $R_{m-1}$, $R_{z}$ and $R_{t}$ relays
$y_{R_{m}}$, $y_{D_{a}}$	Received signals at the $R_{m}$ relay and $D_{a}$ destination
$n_{m}$, $n_{D_{a}}$	AWGN noise at the $R_{m}$ relay and $D_{a}$ destination
$\sigma^{2}$	Noise variance at all the receiver
NF, Bw	Noise figure, transmission bandwidth
Φ, $H\left(.\right)$	Average transmit-power-to-noise-ratio and Heaviside function
$\omega_{u,v}$	Variance of small-scale fading from transmitter *u* to receiver *v*
$\exp\left(.\right)$, $\log\left(.\right)$	Exponential and logarithm functions
$\max\left(.\right)$, $\min\left(.\right)$	Maximum and minimum functions
$F_{X}\left(x\right)$	Cumulative distribution function (CDF) of RV *X*
${\bar{F}}_{X}\left(x\right)$	Complementary Cumulative distribution function (CCDF) of RV *X*
$M_{X}\left(x\right)$	Moment generating function (MGF) of RV *X*
$f_{X}\left(x\right)$	Probability density function (PDF) of RV *X*
OP${}_{a}^{w}$	Outage probability of the $D_{a}$ destination under *w* scheme
PT${}^{w}$	Potential throughput of the whole networks under *w* scheme

## Data Availability

Not applicable.

## Appendix A. Proof of Proposition 1

In this section, the MGF of the sum of N i.n.i.d. exponential RVs are derived. Let us begin with the definition of the MGF as follows:

(A1) $$\begin{array}{cc} M_{S}\left(s\right)= & E\left\{\exp\left(-s\sum_{i=1}^{N}X_{i}\right)\right\}=\int_{x_{1}=0}^{\infty }\cdots \int_{x_{N}=0}^{\infty }\exp\left(-s\sum_{i=1}^{N}x_{i}\right)f_{X_{1},...X_{N}}\left(x_{1},...,x_{N}\right)dx_{1}...dx_{N}\\ \overset{\left(a\right)}{=} & \prod_{i=1}^{N}\int_{x_{i}=0}^{\infty }\exp\left(-sx_{i}\right)f_{X_{i}}\left(x_{i}\right)dx_{i}\overset{\left(b\right)}{=}\prod_{i=1}^{N}\int_{x_{i}=0}^{\infty }\exp\left(-s\sum_{i=1}^{N}x_{i}\right)\left(\frac{1}{\delta_{i}}\right)\exp\left(-\frac{x_{i}}{\delta_{i}}\right)dx_{i}\\ = & \prod_{i=1}^{N}\frac{1}{1+s\delta_{i}}, \end{array}$$

where $E\left\{.\right\}$ is the expectation operator, $\left(a\right)$ is held by employing the independent properties of N RVs, $\left(b\right)$ is obtained by substituting the probability density function (PDF) of RV $X_{i}$, the last equation is derived by computing the integration and we finish the proof here.

## Appendix B. Proof of Theorem 2

In this section, the outage probability of $D_{a}$, $a\in \left\{1,2\right\}$, under the perfect interference cancellation is derived. Let us begin with OP${}_{1}$ as follows:

(A2) $$\begin{array}{cc} {\mathrm{OP}}_{1}^{P}= & \mathrm{Pr}\left\{\log_{2}\left(1+\gamma_{e2e}^{1,P}\right)\leq R_{1}\right\}\overset{\left(a\right)}{=}1-\Xi_{1}\Xi_{2}\Xi_{3},\\ \Xi_{1}= & \prod_{m=2}^{M}\mathrm{Pr}\left\{\frac{\alpha_{1}{\left|h_{m-1,m}\right|}^{2}\frac{\Phi_{m-1}}{\varsigma_{m-1,m}^{F}}}{\alpha_{2}{\left|h_{m-1,m}\right|}^{2}\frac{\Phi_{m-1}}{\varsigma_{m-1,m}^{F}}+\tau_{m}\frac{\Phi_{m}}{\varsigma_{m}^{N}}{\left|h_{m,m}\right|}^{2}+\sum_{t=0}^{m-2}{\left|h_{t,m}\right|}^{2}\frac{\Phi_{t}}{\varsigma_{t,m}^{F}}+1}\geq \gamma_{\mathrm{th}}^{1}\right\},\\ \Xi_{2}= & \mathrm{Pr}\left\{\frac{\alpha_{1}{\left|h_{0,1}\right|}^{2}\frac{\Phi_{0}}{\varsigma_{0,1}^{F}}}{\alpha_{2}{\left|h_{0,1}\right|}^{2}\frac{\Phi_{0}}{\varsigma_{0,1}^{F}}+\tau_{1}\frac{\Phi_{1}}{\varsigma_{1}^{N}}{\left|h_{1,1}\right|}^{2}+1}\geq \gamma_{\mathrm{th}}^{1}\right\},\\ \Xi_{3}= & \mathrm{Pr}\left\{\frac{\alpha_{1}\frac{\Phi_{M}}{\varsigma_{M,D_{1}}^{F}}{\left|h_{M,D_{1}}\right|}^{2}}{\alpha_{2}\frac{\Phi_{M}}{\varsigma_{M,D_{1}}^{F}}{\left|h_{M,D_{1}}\right|}^{2}+\sum_{t=0}^{M-1}\frac{\Phi_{t}}{\varsigma_{t,D_{1}}^{F}}{\left|h_{t,D_{1}}\right|}^{2}+1}\geq \gamma_{\mathrm{th}}^{1}\right\}, \end{array}$$

where $\left(a\right)$ is obtained due to the independence of terms in $\gamma_{e2e}^{1,P}$. $\Xi_{1}$ is computed as

(A3) $$\begin{array}{cc} \Xi_{1}= & \prod_{m=2}^{M}\mathrm{Pr}\left\{\frac{\alpha_{1}{\left|h_{m-1,m}\right|}^{2}\frac{\Phi_{m-1}}{\varsigma_{m-1,m}^{F}}}{\alpha_{2}{\left|h_{m-1,m}\right|}^{2}\frac{\Phi_{m-1}}{\varsigma_{m-1,m}^{F}}+\tau_{m}\frac{\Phi_{m}}{\varsigma_{m}^{N}}{\left|h_{m,m}\right|}^{2}+\sum_{t=0}^{m-2}{\left|h_{t,m}\right|}^{2}\frac{\Phi_{t}}{\varsigma_{t,m}^{F}}+1}\geq \gamma_{\mathrm{th}}^{1}\right\}\\ = & H\left(\Delta_{1}\right)\prod_{m=2}^{M}\exp\left(-f_{1}\left(m\right)\right)\int_{x=0}^{\infty }\int_{y=0}^{\infty }\exp\left(-f_{1}\left(m\right)\tau_{m}\frac{\Phi_{m}}{\varsigma_{m}^{N}}x\right)\exp\left(-f_{1}\left(m\right)y\right)\\ \; & \times f_{X={\left|h_{m,m}\right|}^{2}}\left(x\right)f_{Y=\sum_{t=0}^{m-2}{\left|h_{t,m}\right|}^{2}}\left(y\right)dxdy\\ \overset{\left(a\right)}{=} & H\left(\Delta_{1}\right)\prod_{m=2}^{M}\exp\left(-f_{1}\left(m\right)\right)M_{X}\left(f_{1}\left(m\right)\tau_{m}\frac{\Phi_{m}}{\varsigma_{m}^{N}}\right)M_{Y}\left(f_{1}\left(m\right)\frac{\Phi_{t}}{\varsigma_{t,m}^{F}}\right)\\ \overset{\left(b\right)}{=} & H\left(\Delta_{1}\right)\prod_{m=2}^{M}\exp\left(-f_{1}\left(m\right)\right){\left(1+f_{1}\left(m\right)\tau_{m}\omega_{m,m}\frac{\Phi_{m}}{\varsigma_{m}^{N}}\right)}^{-1}\left[\prod_{o=2}^{m}{\left(1+f_{1}\left(m\right)\frac{\Phi_{o-2}}{\varsigma_{o-2,m}^{F}}\omega_{o-2,m}\right)}^{-1}\right]. \end{array}$$

where $\lambda_{a}=\frac{\gamma_{\mathrm{th}}^{a}}{\Delta_{a}}$, $\Delta_{1}=\alpha_{1}-\alpha_{2}\gamma_{\mathrm{th}}^{1}$ and $\Delta_{2}=\alpha_{2}$, $f_{a}\left(x\right)=\frac{\lambda_{a}\varsigma_{x-1,x}^{F}}{\left(\Phi \omega_{x-1,x}\right)}$, $\gamma_{\mathrm{th}}^{a}=2^{R_{a}}-1$, $\left(a\right)$ is attained by utilizing the definition of MGF function while $\left(b\right)$ is held by employing the outcome of Theorem 1. Similarly, we can straightforwardly derive $\Xi_{2}$ and $\Xi_{3}$ by utilizing the same approach as $\Xi_{1}$ and are given as follows:

(A4) $$\begin{array}{cc} \Xi_{2}= & \mathrm{Pr}\left\{\frac{\alpha_{1}{\left|h_{0,1}\right|}^{2}\frac{\Phi_{0}}{\varsigma_{0,1}^{F}}}{\alpha_{2}{\left|h_{0,1}\right|}^{2}\frac{\Phi_{0}}{\varsigma_{0,1}^{F}}+\tau_{1}\frac{\Phi }{\varsigma_{1}^{N}}{\left|h_{1,1}\right|}^{2}+1}\geq \gamma_{\mathrm{th}}^{1}\right\}\\ = & H\left(\Delta_{1}\right)\exp\left(-f_{1}\left(1\right)\right){\left(1+f_{1}\left(1\right)\tau_{1}\omega_{1,1}\frac{\Phi_{1}}{\varsigma_{1}^{N}}\right)}^{-1},\\ \Xi_{3}= & \mathrm{Pr}\left\{\frac{\alpha_{1}\frac{\Phi_{M}}{\varsigma_{M,D_{1}}^{F}}{\left|h_{M,D_{1}}\right|}^{2}}{\alpha_{2}\frac{\Phi_{M}}{\varsigma_{M,D_{1}}^{F}}{\left|h_{M,D_{1}}\right|}^{2}+\sum_{t=0}^{M-1}\frac{\Phi_{t}}{\varsigma_{t,D_{1}}^{F}}{\left|h_{t,D_{1}}\right|}^{2}+1}\geq \gamma_{\mathrm{th}}^{1}\right\}\\ = & H\left(\Delta_{1}\right)\exp\left(-\frac{\lambda_{1}\varsigma_{M,D_{1}}^{F}}{\Phi \omega_{M,D_{1}}}\right)\prod_{l=1}^{M}{\left(1+\lambda_{1}\frac{\varsigma_{M,D1}^{F}\omega_{l-1,D1}}{\varsigma_{l-1,D1}^{F}\omega_{M,D1}}\right)}^{-1}. \end{array}$$

Having obtained $\Xi_{1},\Xi_{2}$, and $\Xi_{3}$ the OP${}_{1}^{P}$ under the perfect interference cancellation in (A2) is rewritten as

(A5) $$\begin{array}{cc} {\mathrm{OP}}_{1}^{P}= & 1-H\left(\Delta_{1}\right)\left[\exp\left(-\frac{\lambda_{1}\varsigma_{M,D_{1}}^{F}}{\Phi \omega_{M,D_{1}}}\right)\prod_{l=1}^{M}{\left(1+\lambda_{1}\frac{\varsigma_{M,D1}^{F}\omega_{l-1,D1}}{\varsigma_{l-1,D1}^{F}\omega_{M,D1}}\right)}^{-1}\right]\left[\prod_{z=1}^{M}\exp\left(-f_{1}\left(z\right)\right)\right]\\ \; & \times \left[\prod_{i=1}^{M}{\left(1+\tau_{i}\omega_{i,i}\frac{\Phi }{\varsigma_{i}^{N}}\right)}^{-1}\right]\left[\prod_{m=2}^{M}\prod_{o=2}^{m}{\left(1+f_{1}\left(m\right)\frac{\Phi }{\varsigma_{o-2,m}^{F}}\omega_{o-2,m}\right)}^{-1}\right]. \end{array}$$

Now, let us derive the mathematical framework of OP${}_{2}^{P}$ that is given as

(A6) $$\begin{array}{cc} {\mathrm{OP}}_{2}^{P}= & \mathrm{Pr}\left\{\log_{2}\left(1+\gamma_{e2e}^{2,P}\right)\leq R_{2}\right\}=1-\Xi_{4}\Xi_{5}\Xi_{6},\\ \Xi_{4}= & \mathrm{Pr}\left\{\frac{\alpha_{2}{\left|h_{0,1}\right|}^{2}\frac{\Phi_{0}}{\varsigma_{0,1}^{F}}}{\tau_{1}\frac{\Phi_{1}}{\varsigma_{1}^{N}}{\left|h_{1,1}\right|}^{2}+1}\geq \gamma_{\mathrm{th}}^{2}\right\}\\ \Xi_{5}= & \mathrm{Pr}\left\{\frac{\alpha_{2}\frac{\Phi_{M}}{\varsigma_{M,D_{2}}^{F}}{\left|h_{M,D_{2}}\right|}^{2}}{\sum_{t=0}^{M-1}\frac{\Phi_{t}}{\varsigma_{t,D_{2}}^{F}}{\left|h_{t,D_{2}}\right|}^{2}+1}\geq \gamma_{\mathrm{th}}^{2}\right\}\\ \Xi_{6}= & \prod_{m=2}^{M}\mathrm{Pr}\left\{\frac{\alpha_{2}{\left|h_{m-1,m}\right|}^{2}\frac{\Phi_{m-1}}{\varsigma_{m-1,m}^{F}}}{\tau_{m}\frac{\Phi_{m}}{\varsigma_{m}^{N}}{\left|h_{m,m}\right|}^{2}+\sum_{t=0}^{m-2}{\left|h_{t,m}\right|}^{2}\frac{\Phi_{t}}{\varsigma_{t,m}^{F}}+1}\geq \gamma_{\mathrm{th}}^{2}\right\}, \end{array}$$

where the computation of $\Xi_{4},\Xi_{5},\Xi_{6}$ is similar to $\Xi_{1}$ thus, it is omitted here. Finally, by unifying the mathematical framework of both OP, we attain (15) and close the proof here.

## Appendix C. Proof of Theorem 3

The OP performance under the IIC scheme is computed as follows:

(A7) $$\begin{array}{cc} {\mathrm{OP}}_{1}^{I}= & \mathrm{Pr}\left\{\log_{2}\left(1+\gamma_{e2e}^{1,I}\right)\leq R_{1}\right\}\overset{\left(a\right)}{=}1-\Psi_{1}\Xi_{3},\\ \Psi_{1}= & \prod_{m=1}^{M}\mathrm{Pr}\left\{\frac{\alpha_{1}{\left|h_{m-1,m}\right|}^{2}\frac{\Phi_{m-1}}{\varsigma_{m-1,m}^{F}}}{\alpha_{2}{\left|h_{m-1,m}\right|}^{2}\frac{\Phi_{m-1}}{\varsigma_{m-1,m}^{F}}+\tau_{m}\frac{\Phi_{m}}{\varsigma_{m}^{N}}{\left|h_{m,m}\right|}^{2}+\sum_{t=0,t\neq \left\{m-1,m\right\}}^{M-1}{\left|h_{t,m}\right|}^{2}\frac{\Phi_{t}}{\varsigma_{t,m}^{F}}+1}\geq \gamma_{\mathrm{th}}^{1}\right\}\\ = & H\left(\Delta_{1}\right)\prod_{m=2}^{M-1}\exp\left(-f_{1}\left(m\right)\right)\int_{x=0}^{\infty }\int_{z=0}^{\infty }\exp\left(-f_{1}\left(m\right)\tau_{m}\frac{\Phi_{m}}{\varsigma_{m}^{N}}x\right)\\ \; & \times \exp\left(-f_{1}\left(m\right)\frac{\Phi }{\varsigma_{z,m}^{F}}z\right)f_{X={\left|h_{m,m}\right|}^{2}}\left(x\right)f_{Z=\sum_{z=0,z\neq \left\{m-1,m\right\}}^{M-1}{\left|h_{z,m}\right|}^{2}}\left(z\right)dxdz\\ \overset{\left(a\right)}{=} & H\left(\Delta_{1}\right)\prod_{m=2}^{M-1}\exp\left(-f_{1}\left(m\right)\right)M_{X}\left(f_{1}\left(m\right)\tau_{m}\frac{\Phi }{\varsigma_{m}^{N}}\right)M_{Z}\left(f_{1}\left(m\right)\frac{\Phi }{\varsigma_{z,m}^{F}}\right)\\ \overset{\left(b\right)}{=} & H\left(\Delta_{1}\right)\prod_{m=2}^{M-1}\exp\left(-f_{1}\left(m\right)\right){\left(1+f_{1}\left(m\right)\tau_{m}\omega_{m,m}\frac{\Phi_{m}}{\varsigma_{m}^{N}}\right)}^{-1}\\ \; & \times \left[\prod_{z=0,z\neq \left\{m-1,m\right\}}^{M-1}{\left(1+f_{1}\left(m\right)\frac{\Phi_{z}}{\varsigma_{z,m}^{F}}\omega_{z,m}\right)}^{-1}\right]. \end{array}$$

Similarly, the ${\mathrm{OP}}_{2}^{I}$ is then computed as follows:

(A8) $$\begin{array}{cc} {\mathrm{OP}}_{2}^{I}= & \mathrm{Pr}\left\{\log_{2}\left(1+\gamma_{e2e}^{2,I}\right)\leq R_{2}\right\}\overset{\left(a\right)}{=}1-\Psi_{2}\Psi_{3},\\ \Psi_{2}= & \prod_{m=1}^{M}\;\mathrm{Pr}\;\left\{\;\frac{\alpha_{2}{\left|h_{m-1,m}\right|}^{2}\frac{\Phi_{m-1}}{\varsigma_{m-1,m}^{F}}}{\epsilon_{m}\alpha_{1}{\left|h_{m-1,m}\right|}^{2}\frac{\Phi_{m-1}}{\varsigma_{m-1,m}^{F}}+\tau_{m}\frac{\Phi_{m}}{\varsigma_{m}^{N}}{\left|h_{m,m}\right|}^{2}+\sum_{t=0,t\neq \left\{m-1,m\right\}}^{M-1}{\left|h_{t,m}\right|}^{2}\frac{\Phi_{t}}{\varsigma_{t,m}^{F}}+1}\;\geq \;\gamma_{\mathrm{th}}^{2}\;\right\}\\ = & H\left(\Delta_{3}\right)\prod_{m=1}^{M}\exp\left(-f_{3}\left(m\right)\right)\int_{x=0}^{\infty }\int_{z=0}^{\infty }\exp\left(-f_{3}\left(m\right)\tau_{m}\frac{\Phi_{m}}{\varsigma_{m}^{N}}x\right)\\ \; & \times \exp\left(-f_{3}\left(m\right)z\frac{\Phi }{\varsigma_{z,m}^{F}}\right)f_{X={\left|h_{m,m}\right|}^{2}}\left(x\right)f_{Z=\sum_{z=0,z\neq \left\{m-1,m\right\}}^{M-1}{\left|h_{z,m}\right|}^{2}}\left(z\right)dxdz\\ \overset{\left(a\right)}{=} & H\left(\Delta_{3}\right)\prod_{m=1}^{M}\exp\left(-f_{3}\left(m\right)\right){\left(1+f_{3}\left(m\right)\tau_{m}\omega_{m,m}\frac{\Phi_{m}}{\varsigma_{m}^{N}}\right)}^{-1}\\ \; & \times \left[\prod_{z=0,z\neq \left\{m-1,m\right\}}^{M}{\left(1+f_{3}\left(m\right)\frac{\Phi_{z}}{\varsigma_{z,m}^{F}}\omega_{z,m}\right)}^{-1}\right]\\ \Psi_{3}= & \mathrm{Pr}\left\{\frac{\alpha_{2}\frac{\Phi_{M}}{\varsigma_{M,D_{2}}^{F}}{\left|h_{M,D_{2}}\right|}^{2}}{\epsilon_{D_{2}}\alpha_{1}\frac{\Phi_{M}}{\varsigma_{M,D_{1}}^{F}}{\left|h_{M,D_{1}}\right|}^{2}+\sum_{t=0}^{M-1}\frac{\Phi_{t}}{\varsigma_{t,D_{2}}^{F}}{\left|h_{t,D_{2}}\right|}^{2}+1}\geq \gamma_{\mathrm{th}}^{2}\right\}\\ = & H\left(\Delta_{2}\right){\left(1+\lambda_{2}\epsilon_{D_{2}}\alpha_{1}\frac{\varsigma_{M,D_{2}}^{F}\omega_{M,D_{1}}}{\varsigma_{M,D_{1}}^{F}\omega_{M,D_{2}}}\right)}^{-1}\exp\left(-\frac{\lambda_{2}\varsigma_{M,D_{2}}^{F}}{\Phi \omega_{M,D_{2}}}\right)\prod_{l=1}^{M}{\left(1+\lambda_{2}\frac{\varsigma_{M,D_{2}}^{F}\omega_{l-1,D_{2}}}{\varsigma_{l-1,D_{2}}^{F}\omega_{M,D_{2}}}\right)}^{-1} \end{array}$$

where $\Delta_{3}=\alpha_{2}-\epsilon_{m}\alpha_{1}\gamma_{\mathrm{th}}^{2}$, $\upsilon =\frac{\gamma_{\mathrm{th}}^{2}}{\Delta_{3}}$ and $f_{3}\left(x\right)=\frac{\upsilon \varsigma_{x-1,x}^{F}}{\Phi_{x-1}\omega_{x-1,x}}$.

## Appendix D. Proof of Proposition 1

In this appendix, we are going to derive the behaviors of OP with respect to the total transmit power. Particularly, we first derive the ${\mathrm{OP}}_{a}^{P}$ as a function of $P_{\mathrm{tot}}$. Let us rewrite it explicitly as a function of $\Phi =\frac{P_{\mathrm{tot}}}{\sigma^{2}\left(M+1\right)}=\frac{P}{\sigma^{2}}=x$ as follows:

(A9) $$\begin{array}{cc} {\mathrm{OP}}_{a}^{P}\left(x\right)= & 1-V_{1}\exp\left(-\frac{\lambda_{a}\varsigma_{M,D_{a}}^{F}}{x\omega_{M,D_{a}}}\right)\prod_{v=1}^{M}\exp\left(-f_{a}\left(v,x\right)\right), \end{array}$$

where $V_{1}=H\left(\Delta_{a}\right)\left[\prod_{i=1}^{M}{\left(1+\tau_{i}\omega_{i,i}\frac{1}{\varsigma_{i}^{N}}\right)}^{-1}\right]$$\times \left[\prod_{m=2}^{M}\prod_{o=2}^{m}{\left(1+f_{a}\left(m\right)\frac{1}{\varsigma_{o-2,m}^{F}}\omega_{o-2,m}\right)}^{-1}\right]\left[\prod_{l=1}^{M}{\left(1+\lambda_{a}\frac{\varsigma_{M,D_{a}}^{F}\omega_{l-1,D_{a}}}{\varsigma_{l-1,D_{a}}^{F}\omega_{M,D_{a}}}\right)}^{-1}\right]$. Let us take the first-order derivative of OP${}_{a}^{P}$ with respect to *x* as follows:

(A10) $$\begin{array}{cc} {\overset{•}{\mathrm{OP}}}_{a}\left(x\right)= & \;-\;\frac{V_{1}}{x^{2}}\left[\prod_{v=1}^{M}\exp\left(-f_{a}\left(v,x\right)\right)\right]\exp\left(-\frac{\lambda_{a}\varsigma_{M,D_{a}}^{F}}{x\omega_{M,D_{a}}}\right)\left[\frac{\lambda_{a}\varsigma_{M,D_{a}}^{F}}{\omega_{M,D_{a}}}+\sum_{v=1}^{M}\left(\frac{\lambda_{a}\varsigma_{v-1,v}^{F}}{\omega_{v-1,v}}\right)\right]\;<\;0\;. \end{array}$$

From (A10), it is obvious that OP is a monotonic decrease function with respect to *x*. Direct inspection (13), it is obvious that the impact of $P_{\mathrm{tot}}$ on the PT relied only on the OP thus, PT monotonically increases with $P_{\mathrm{tot}}$. Direct inspection (16), we observe that the framework of ${\mathrm{OP}}_{a}^{I}$ with respect to $P_{\mathrm{tot}}$ is similar to the ${\mathrm{OP}}_{a}^{P}$, thus, it has the same trend as ${\mathrm{OP}}_{a}^{P}$ so we skip the derivation and finish the proof here.
